# Glucose-dependent GPER1 expression modulates tamoxifen-induced IGFBP-1 accumulation

**DOI:** 10.1530/JME-18-0253

**Published:** 2019-05-29

**Authors:** Yan Zheng, Kevin D Houston

**Affiliations:** 1 Department of Chemistry and Biochemistry, New Mexico State University, Las Cruces, New Mexico, USA

**Keywords:** breast cancer, estrogen receptors, IGFBP-1, metabolism

## Abstract

G protein-coupled estrogen receptor 1 (GPER1) is a seven-transmembrane receptor that mediates rapid cell signaling events stimulated by estrogens. While the role that GPER1 has in the modulation of E2-responsive tissues and cancers is well documented, the molecular mechanisms that regulate GPER1 expression are currently not well defined. The recently identified GPER1-dependent mechanism of tamoxifen action in breast cancer cells underscores the importance of identifying mechanisms that regulate GPER1 expression in this cell type. We hypothesized that GPER1 expression in breast cancer cells is sensitive to [D-glucose] and provide data showing increased GPER1 expression when cells were cultured in low [D-glucose]. To determine if the observed accumulation of GPER1 was AMP-activated protein kinase (AMPK)-dependent, small molecule stimulation or inhibition of AMPK was performed. AMPK inhibition decreased GPER1 accumulation in cells grown in low [D-glucose] while the AMPK-activating compound AICAR increased GPER1 accumulation in cells grown in high [D-glucose] media. Additionally, transfection of cells with a plasmid expressing constitutively active AMPK resulted in increased GPER1 accumulation. To determine if [D-glucose]-dependent GPER1 accumulation altered breast cancer cell response to tamoxifen, cells grown in the presence of decreasing [D-glucose] were co-treated with tamoxifen and IGFBP-1 transcription was measured. The results from these experiments reveal that D-glucose deprivation increased GPER1-mediated and tamoxifen-induced IGFBP-1 transcription suggesting that [D-glucose] may increase breast cancer cell sensitivity to tamoxifen. Taken together, these results identify a previously unknown mechanism that regulates GPER1 expression that modifies one aspect tamoxifen action in breast cancer cells.

## Introduction

Estrogen is a critical signaling molecule that modulates many cell signaling pathways and physiological functions. The classical mechanism by which estrogen signals in cells is via binding to estrogen receptor (ER), inducing conformational change of this transcription factor, subsequent association with estrogen response elements (ERE) within the promoters of target genes thus promoting gene transcription (Klinge 2001). More recently, non-classical mechanisms of estrogen signaling mediated by membrane-associated ER or the G protein-coupled estrogen receptor 1 (GPER1, aka GPR30) have been identified (Carmeci *et al.* 1997, Kvingedal & Smeland 1997, Takada *et al.* 1997, Filardo & Thomas 2012). The presence of non-classical estrogen signaling mechanisms in cells has resulted in a need to understand the role that these pathways play in physiology and disease.

Both *in vitro* and *in vivo* studies have provided evidence for the importance of the non-classical estrogen receptor GPER1 in both normal and disease states. A recurring theme in these studies is the observation of the loss of metabolic homeostasis in GPER1 knockout (KO) mice. For example, accumulation of visceral fat and increased body weight (Haas *et al.* 2009, Ford *et al.* 2011), as well as abnormal perivascular and subcutaneous adiposity were observed in GPER1 KO mice (Meyer *et al.* 2013, Sharma *et al.* 2013). Additionally, the GPER1-specific agonist G1 inhibited lipogenesis in both human and rodent pancreatic islets (Tiano *et al.* 2011). Beyond the observed dysregulation of adipogenesis, others reported that male GPER1 KO mice developed hyperinsulinemia and hyperglycemia by 18 months of age (Sharma *et al.* 2013) and female GPER1 KO mice developed impaired glucose tolerance within 6 months of age (Martensson *et al.* 2009). Of note, both classical and non-classical estrogen signaling regulates glucose uptake, glucose storage, insulin secretion and has a role in insulin sensitivity (Brown & Clegg 2010, Barton & Prossnitz 2015). Contrary to these findings, observations from one study showed that GPER1 KO mice had increased glucose homeostasis and reduced non-fasting and fasting blood glucose concentrations (Wang *et al.* 2016). Taken together, observations from multiple studies indicate a central role for GPER1 in metabolic homeostasis.

It is well known that gene expression is sensitive to [D-glucose] and this regulation is mediated by putative glucose responsive elements (5′-CANNTG-XXXXX-CANNTG-3′) in the promoters of target genes (Foufelle *et al.* 1998). For example, high [D-glucose] increases the expression of fibroblast growth factor 21 (FGF21) in rat hepatocytes (Iizuka *et al.* 2009) and zinc finger transcription factor Egr-1 in pancreatic cells (Johnston 1999). D-glucose deprivation can also induce the expression of genes such as heme oxygenase-1 (Chang *et al.* 2002) and interleukin 6 (Choi *et al.* 2013). Furthermore, in D-glucose-deprived cells, AMP-activated protein kinase (AMPK) mediates the induction of multiple genes via activation of several transcription factors (Canto & Auwerx 2010). Of interest in breast cancer cells, D-glucose deprivation enhances the sensitivity of breast cancer cells to tamoxifen (Ambrosio *et al.* 2017) suggesting that the low [D-glucose] modulates the expression of genes involved in tamoxifen action in these cells. Our previous work identified GPER1 as a key mediator of tamoxifen action in breast cancer cells (Vaziri-Gohar & Houston 2016) leading to the hypothesis that GPER1 expression is sensitive to [D-glucose] and changes in GPER1 expression alters the tamoxifen response in breast cancer cells.

In this study, GPER1 expression was significantly induced upon D-glucose deprivation in tamoxifen-sensitive breast cancer cell lines and the observed increase in GPER1 expression was AMPK-dependent. The impact that [D-glucose] had on GPER1-dependent tamoxifen action in breast cancer cells was demonstrated by measuring the accumulation of IGFBP-1 expression in tamoxifen-treated breast cancer cells in the presence of increasing [D-glucose]. These measurements revealed that in cells exposed to high [D-glucose], IGFBP-1 gene transcription was significantly decreased. Collectively, our data revealed a novel mechanism that regulates GPER1 expression resulting in altered sensitivity of breast cancer cells to tamoxifen.

## Materials and methods

### Cell culture

MCF-7 and T-47D breast cancer cells were purchased from ATCC and were cultured in maintenance DMEM supplemented with 10% fetal bovine serum, 1 mM sodium pyruvate and 2 mM L-glutamine (Life Technologies). All cell lines were maintained in maintenance media and cells for experiments were within passages 35. Eker rat uterine leiomyoma cells ELT-3 and ELT-6 were kindly provided by Cheryl Lyn Walker (Baylor College of Medicine, Houston, TX) and cultured in maintenance DF-8 media as described before (Howe *et al.* 1995).

### Cell treatment

For D-glucose deprivation, maintenance media were removed from cells growing in logarithmic phase, washed with 1× PBS, and replaced with serum-free, D-glucose-free DMEM and the indicated concentrations of D-glucose was added. Cells were treated with the indicted dose of Compound C (Abcam) after washing with 1× PBS and were glucose-starved in D-glucose-free and serum-free DMEM for 24 h. For the dorsomorphin (Compound C) (Abcam) treatment, dimethyl sulfoxide (DMSO) was used as solvent and cells were washed with 1× PBS followed by treatment with the indicated amount of dorsomorphin in D-glucose free DMEM for 24 h. For the 5-Aminoimidazole-4-carboxamide ribonucleotide (AICAR) (Abcam) treatment, dd H_2_O was used as solvent. Cells were washed with 1× PBS and treated with serum-free DMEM containing 25 mM D-glucose for 24 h. Cells were then washed with 1× PBS followed by treatment with the indicated amount of AICAR in serum-free DMEM containing 25 mM D-glucose for 24 h. 4-hydroxytamoxifen (Tam) (Fluka, St. Louis, MO) treatment was described before (Vaziri-Gohar & Houston 2016). Briefly, 48 h prior to the treatment, MCF-7 and T-47D cells were washed with 1× PBS and serum-starved with phenol red-free DMEM supplemented with 1% charcoal-stripped FBS (Life Technologies). After 24 h, cells were washed with 1× PBS and treated with indicated concentrations of Tam in serum-free DMEM for 24 h. Ethanol was used to dissolve Tam. After treatments, cells were collected for either qRT-PCR or immunoblot analysis. For Tam and G-36 (Cayman Chemical) co-treatment, MCF-7 and T-47D cells were washed with 1× PBS and cultured in phenol red-free DMEM supplemented with 1% charcoal-stripped FBS (Life Technologies) for 48 h followed by 24 h of treatment with 1 µM Tam and 1 µM G-36 in serum-free DMEM for 24 h. After treatment, cells were collected for either qRT-PCR or immunoblot analysis.

### Plasmid transfection

Plasmid pEBG‐AMPK α1(1‐312) and plasmid pEBG were purchased from Addgene (Addgene, Cambridge, MA) and all cell transfections were performed using Lipofectamine 2000 reagent in serum-free Opti-MEM (Life Technologies). After 6 h of transfection, cells were washed with 1× PBS and cultured in DMEM containing 25 mM D-glucose and 10%FBS 48 h prior to sample collection for immunoblot analysis.

### Total RNA extraction and quantitative real-time PCR analysis

Total RNA was extracted and isolated with the PureLink RNA Mini Kit (Life Technologies) followed by on-column DNA digestion using Purelink DNase Set (Life Technologies). cDNA was synthesized from 1 μg total RNA using the High Capacity RNA-to-cDNA Kit (Life Technologies) and used as template in subsequent quantitative real-time PCR (qRT-PCR) reactions. qRT-PCR was performed using SYBR Green Master Mix (Life Technologies) and the 7300 Real-Time PCR system (Bio-Rad). Primer pairs used for qRT-PCR: human GPER1 forward 5′-AGT-CGG-ATG-TGA-GGT-TCA-3′; reverse 5′-TCT-GTG-TGA-GGA-GTG-CAA-3′; human IGFBP-1 forward 5′-CTA-TGA-TGG-CTC-GAA-GGC-TC-3′; reverse 5′-TTC-TTG-TTG-CAG-TTT-GGC-AG-3′ (Xie 2014). Human RPL30 gene was used as the internal control to normalize for mRNA in qRT-PCR reactions. Human RPL30 forward 5′-ACA-GCA-TGC-GGA-AAA-TAC-TAC-3′; reverse 5′-AAA-GGA-AAA-TTT-TGC-AGG-TTT-3′ (de Jonge *et al.* 2007). Cq values for RPL30 qRT-PCR reaction presented in this manuscript are provided in Supplementary Fig. 3 (see section on supplementary data given at the end of this article).

### Immunoblot analysis

To prepare samples for immunoblot analysis, cells were harvested with RIPA lysis buffer containing protease and phosphatase inhibitor cocktails (87785, 78420, Thermo Scientific). After lysis, cells were centrifuged at 12,000 g for 15 min at 4°C, supernatant was collected and protein concentration was determined by BCA assay (Thermo Scientific). About 30–75 μg total protein was resolved using a Bolt 4–12% Bis-Tris Plus gels and transferred to PVDF membrane (Life Technologies). PVDF membranes were blocked in 1× Tris-buffered saline-0.1% Tween 20 (TBST) containing 5% fat-free milk at room temperature for 1 h with slow agitation. Membranes were then washed with 1× TBST three times and primary antibody was added and allowed to incubate overnight at 4°C. The following primary antibodies including dilution factor in 5% milk TBST were used in the current study: GPER1 (sc-48825-R, Santa Cruz Biotechnology) 1:1000; β-actin (sc-47778, Santa Cruz Biotechnology)1;2000; phospho-AMPKα (Thr 172) (#2531, Cell Signaling Technology) 1:500; AMPK (#2532, Cell Signaling Technology) 1:500. After primary antibody incubation, membranes were washed three times with 1× TBST then incubated with anti-mouse IgG conjugated to horseradish peroxidase (sc-81178, Santa Cruz Biotechnology) 1:5000 at room temperature for 1 h. After washing membranes with 1× TBST three times, chemiluminescence reagent (34076, Thermo Scientific) was added and detected using Gel Doc™ XR ChemiDoc™ imaging system (Bio-Rad) followed by quantification using ImageJ (NIH). Restore plus western blot buffer (46430, Thermo Scientific) was used to strip membranes of antibodies prior to probing for loading control where needed.

### Extracellular IGFBP-1 analysis

The method was previously described (Vaziri-Gohar & Houston 2016). Briefly, media was collected and concentrated with centrifugal filter units (UFC800396, MilliporeSigma, Burlington, MA) at 4°C with the speed of 4000 rpm for 1 h. Concentrated media was collected with an addition of protease inhibitor cocktail (Prod #1862209, Thermo Scientific). Total protein concentration of concentrated media was measured by BCA assay, and the level of extracellular IGFBP-1 was determined by immunoblot analysis as previously described. For the external loading control, same amount of total protein (30 µg) of concentrated media samples were resolved by Bolt 4–12% Bis-Tris Plus gels. The gels were then washed with deionized water for 5 min and stained with Coomassie blue for 1 h. Thereafter, gels were de-stained with deionized water overnight. Gels were then imaged with FOTODYNE gel imager (FOTODYNE Incorporated, Hartland, WI).

### Statistical analysis

All statistical analysis was performed by one-way or repeated measures ANOVA for real-time PCR and immunoblot, respectively using Prism 6 (GraphPad). Differences in values were defined as significant if *P* ≤ 0.05. Error bars represent ± S.E.M.

## Results

### GPER1 expression is inversely related to [D-glucose] and correlates with AMPK activation in breast cancer cells

**Figure 1:**
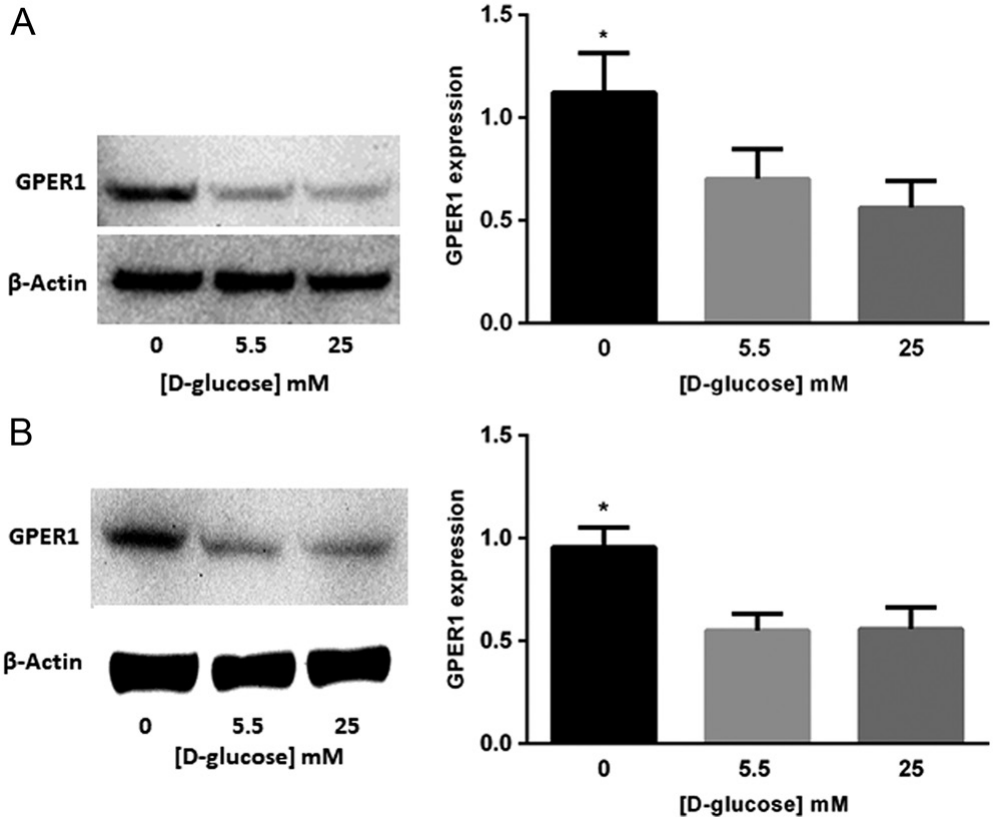
GPER1 expression is elevated in breast cancer cells cultured in low D-glucose-conditions. Immunoblot analysis of GPER1 expression in (A), MCF-7 cells and (B), T-47D cells cultured for 24 h in media containing the indicated [D-glucose]. Results are the average of at least three independent experiments and GPER1 expression was normalized to β-actin. Error bars are the standard error of the mean and statistical significance (*P* < 0.05) is noted using *.

**Figure 2:**
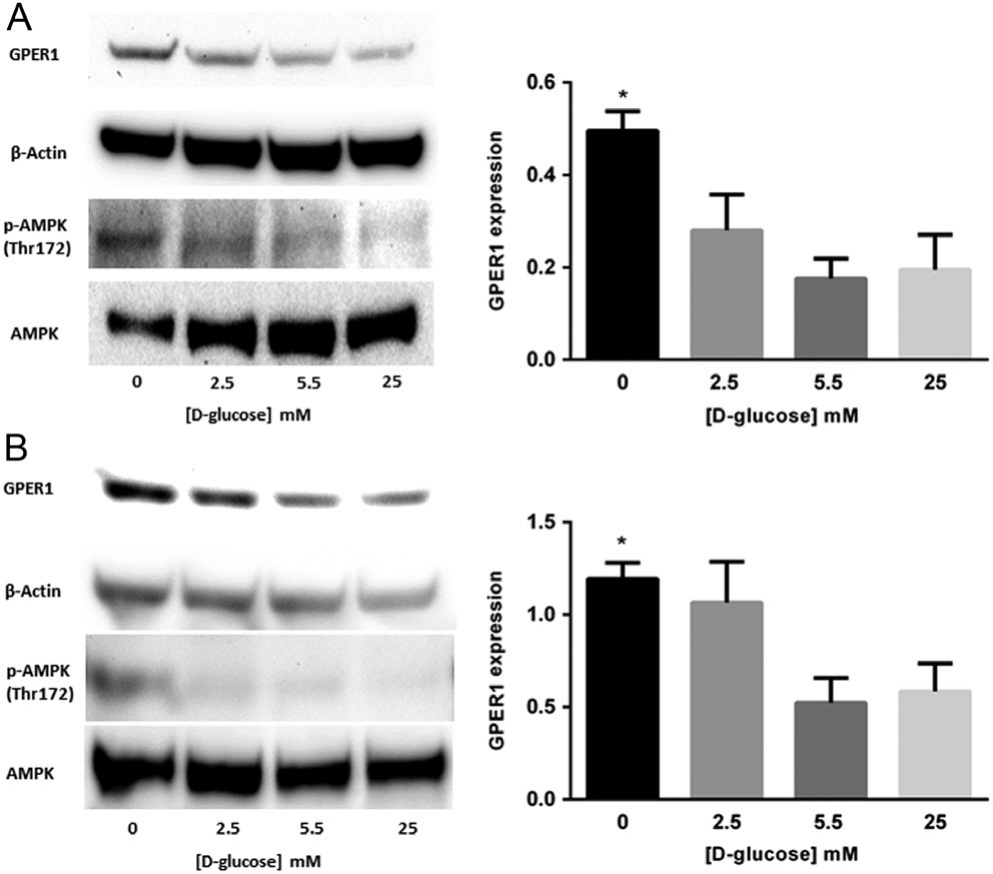
GPER1 and phospho-AMPKα (Thr 172) accumulates in breast cancer cells cultured in low D-glucose conditions. Immunoblot analysis of GPER1 and pAMPK (Thr 172) accumulation in (A), MCF-7 cells and (B), T-47D cells cultured for 24 h in media containing the indicated [D-glucose]. Results are the average of three independent experiments and GPER1 expression was normalized to β-actin. Error bars are the standard error of the mean and statistical significance (*P* < 0.05) is noted using *.

**Figure 3:**
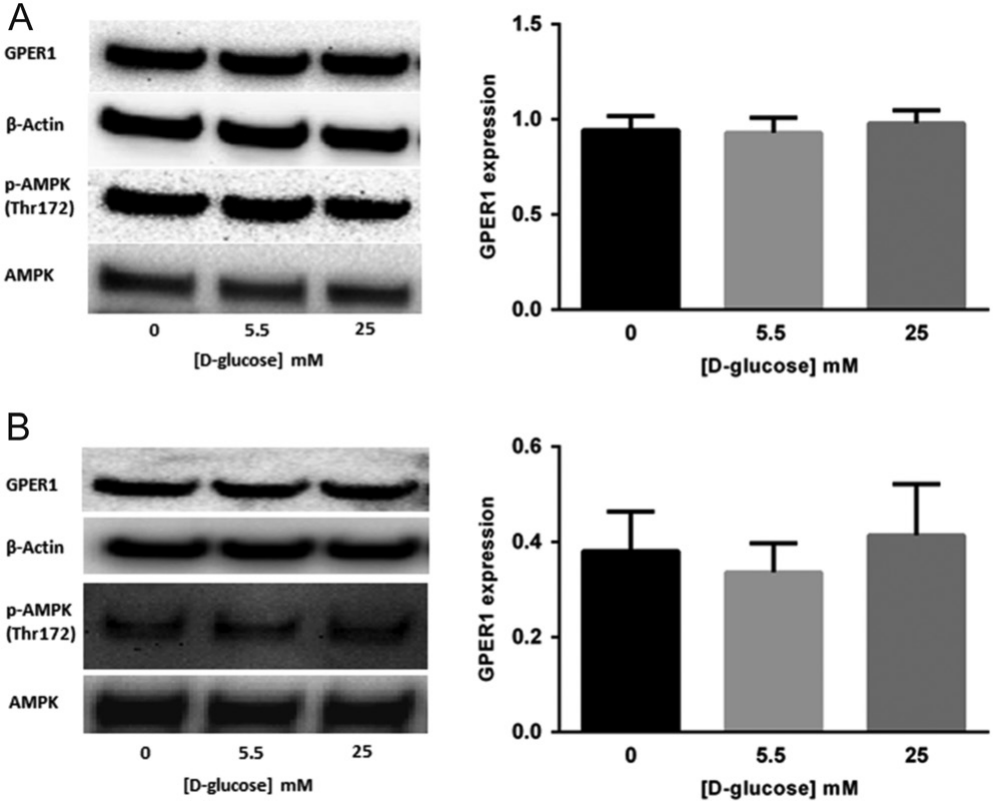
D-glucose deprivation did not significantly change GPER1 expression or pAMPK (Thr 172) accumulation in ELT-3 or ELT-6 cells. Immunoblot analysis of GPER1 and pAMPK (Thr 172) accumulation in (A) ELT-3 cells and (B) ELT-6 cells cultured for 24 h in media containing the indicated [D-glucose]. Results are the average of three independent experiments and GPER1 expression was normalized to β-actin. Error bars are the standard error of the mean.

To determine if GPER1 expression is sensitive to [D-glucose] in breast cancer cells, MCF-7 and T-47D cell lines were grown for 24 h in media containing 0, 5.5 or 25 mM D-glucose and the expression of GPER1 was determined by immunoblot. In the two cell lines tested, the highest levels of GPER1 protein were observed in cells exposed to the lowest [D-glucose] (Fig. 1A and B). In addition, the expression level of GPER1 was compared between MCF-7 and T-47D cells and results indicated that GPER1 expression was similar in these two cell lines when cultured in maintenance media (Supplementary Fig. 1). Additionally, when grown in 10% FBS, the affect was of D-glucose deprivation on GPER1 accumulation was diminished (Supplementary Fig. 2). These data suggested that GPER1 expression was regulated by the [D-glucose] in tamoxifen-sensitive breast cancer cells. The AMPK functions as an energy sensor in cells and is activated when the [AMP]/[ATP] ratio is high (Canto & Auwerx 2010), a phenomenon associated with D-glucose deprivation and indicated by phosphorylation of Thr 172 (Hawley *et al.* 1996, Kim *et al.* 2014). To demonstrate that the growth conditions associated with increased GPER1 expression in breast cancer cells also resulted in increased AMPK activation, MCF-7 and T-47D cells were grown in serum-free media containing to 0, 2.5, 5.5 or 25 mM D-glucose for 24 h followed by immunoblot analysis of GPER1, pAMPK (Thr 172) and AMPK protein levels. In both cell lines, GPER1 levels increased when the [D-glucose] concentration was low and the accumulation of pAMPK (Thr 172) was observed (Fig. 2A and B). To provide further evidence for the AMPK-dependence of the observed increase in GPER1 expression upon D-glucose deprivation, experiments to determine if GPER1 expression is sensitive to [D-glucose] in two Eker rat uterine leiomyoma cell lines (ELT-3 and ELT-6) were performed. ELT-3 and ELT-6 cells are *Tsc2* null resulting in constitutive AMPK activation (Short *et al.* 2008). High [D-glucose] will not inactivate AMPK in these cell lines due to genetic background and thus will not reduce GPER1 expression as observed in breast cancer cell lines. ELT-3 and ELT-6 cells were grown in serum-free media containing 0, 5.5 or 25 mM D-glucose for 24 h followed by immunoblot analysis of GPER1, pAMPK (Thr 172) and AMPK protein levels. We observed no significant decrease in GPER1 expression or pAMPK accumulation in the presence of 25 mM D-glucose in ELT-3 or ELT-6 cells (Fig. 3A and B). Taken together, these data suggest that GPER1 expression in cells deprived of D-glucose was mediated by an AMPK-dependent pathway and this mechanism of regulation for GPER1 expression is not species or tissue specific.

### Specific activation or inhibition of AMPK using small molecules indicates that [D-glucose]-sensitive GPER1 expression is AMPK-dependent

**Figure 4:**
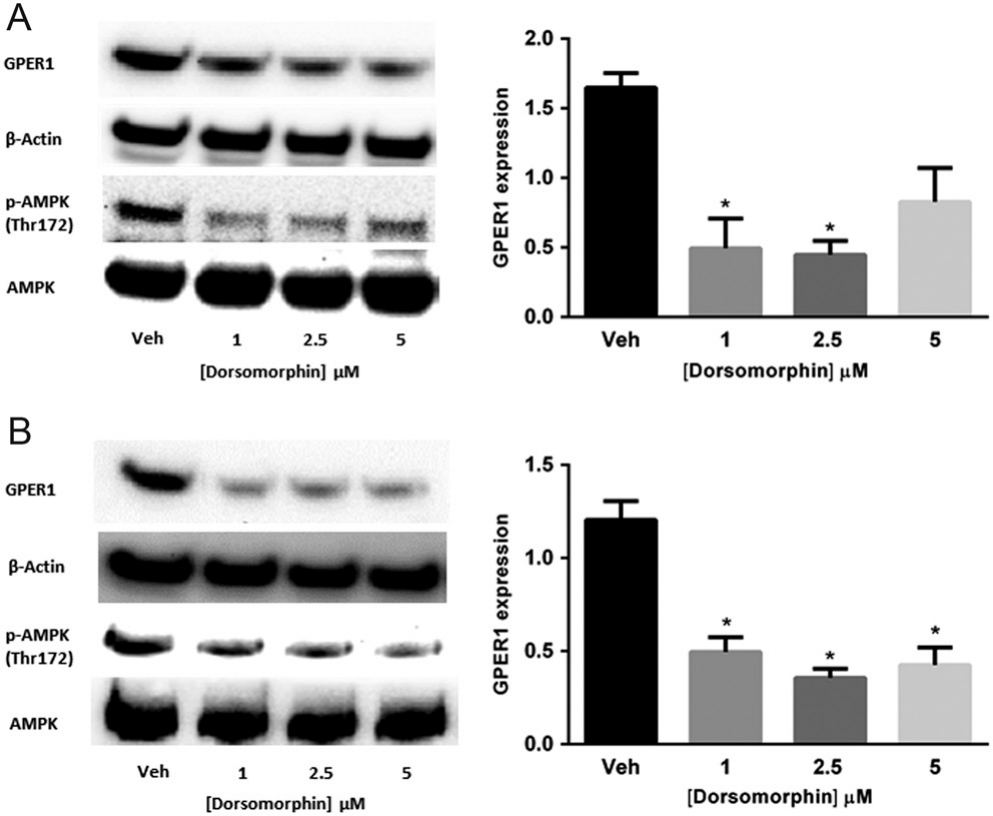
Reduced GPER1 expression observed after AMPK inhibition in ELT-3 and ELT-6 cells. Immunoblot analysis of GPER1 and pAMPK (Thr 172) accumulation in (A) ELT-3 cells and (B) ELT-6 cells cultured for 24 h in media containing the indicated dose of dorsomorphin. Results are the average of three independent experiments and GPER1 expression was normalized to β-actin. Error bars are the standard error of the mean and statistical significance (*P* < 0.05) is noted using *.

**Figure 5:**
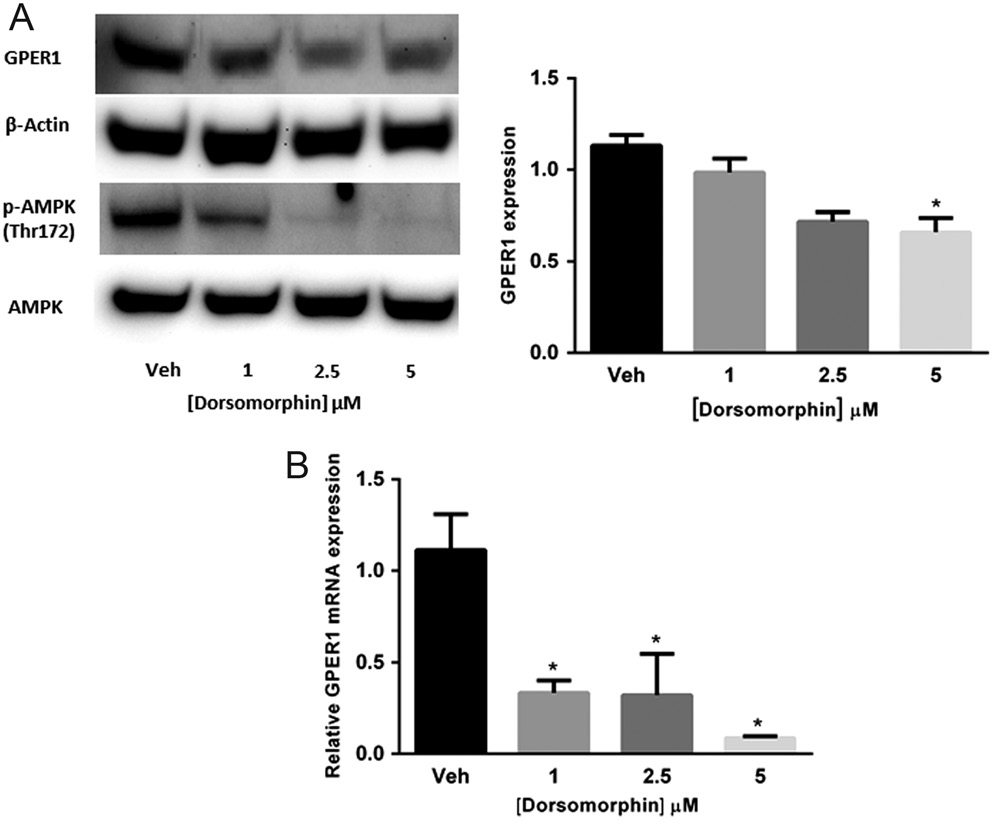
AMPK inhibition decreased GPER1 expression in MCF-7 cells cultured in low [D-glucose]. (A) Immunoblot analysis GPER1 expression and (B) quantitative real-time PCR analysis of GPER1 transcript level in MCF-7 cells cultured in low [D-glucose] after 24-h treatment with the indicated dose of dorsomorphin. Results are the average of 3 independent experiments. GPER1 protein expression was normalized using β-actin and GPER1 transcript levels were normalized using RPL30. Error bars are the standard error of the mean and statistical significance (*P* < 0.05) is noted using *.

**Figure 6:**
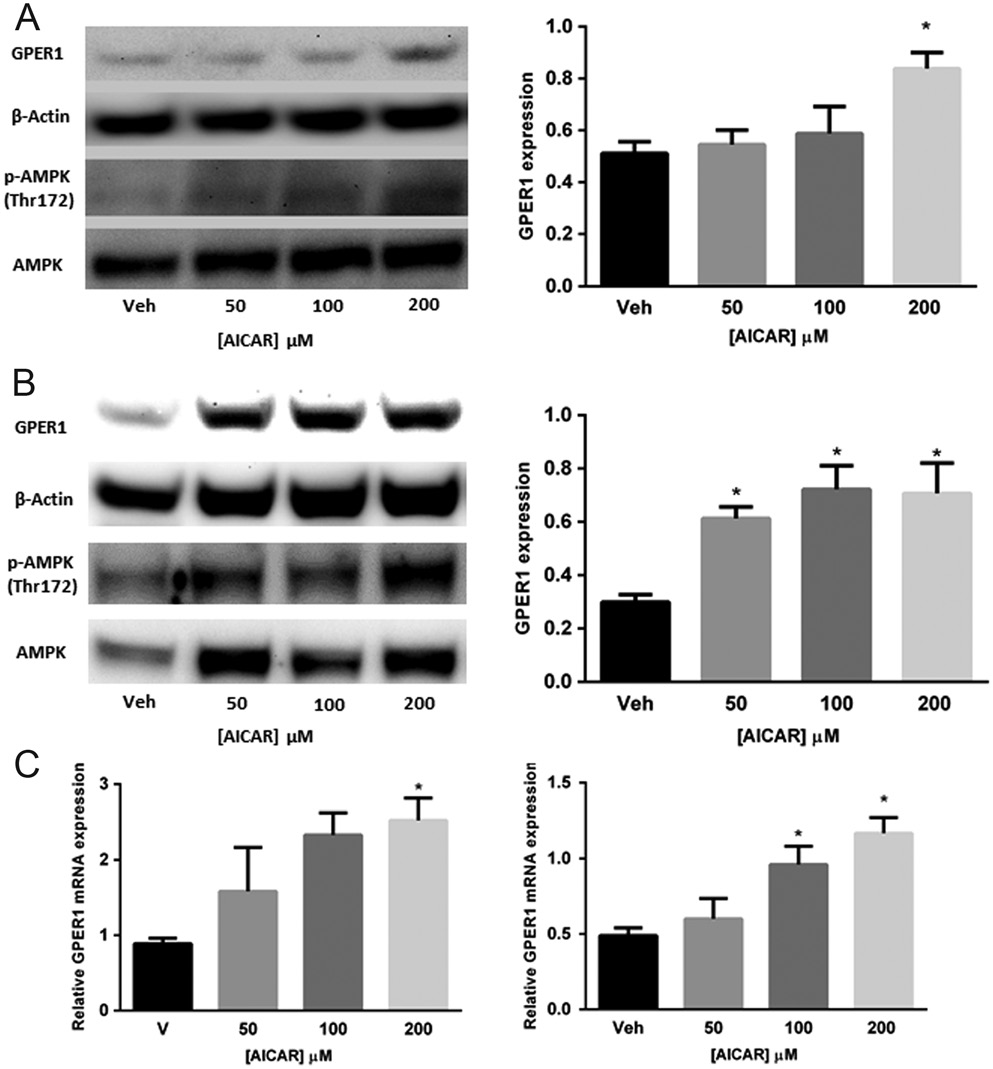
AMPK activation induced GPER1 expression in breast cancer cells. Immunoblot analysis of GPER1 and pAMPK (Thr 172) accumulation in (A) MCF-7 cells and (B) T-47D cells cultured in 25 mM D-glucose and treated for 24 h with the indicated dose of AICAR. (C) Quantitative real-time PCR analysis of GPER1 transcript level in MCF-7 (left) and T-47D (right) cells cultured in 25 mM D-glucose and treated for 24 h with the indicated dose of AICAR. Quantitative results are the average of three independent experiments. GPER1 protein expression was normalized using β-actin and GPER1 transcript levels were normalized using RPL30. Error bars are the standard error of the mean and statistical significance (*P* < 0.05) is noted using *.

**Figure 7:**
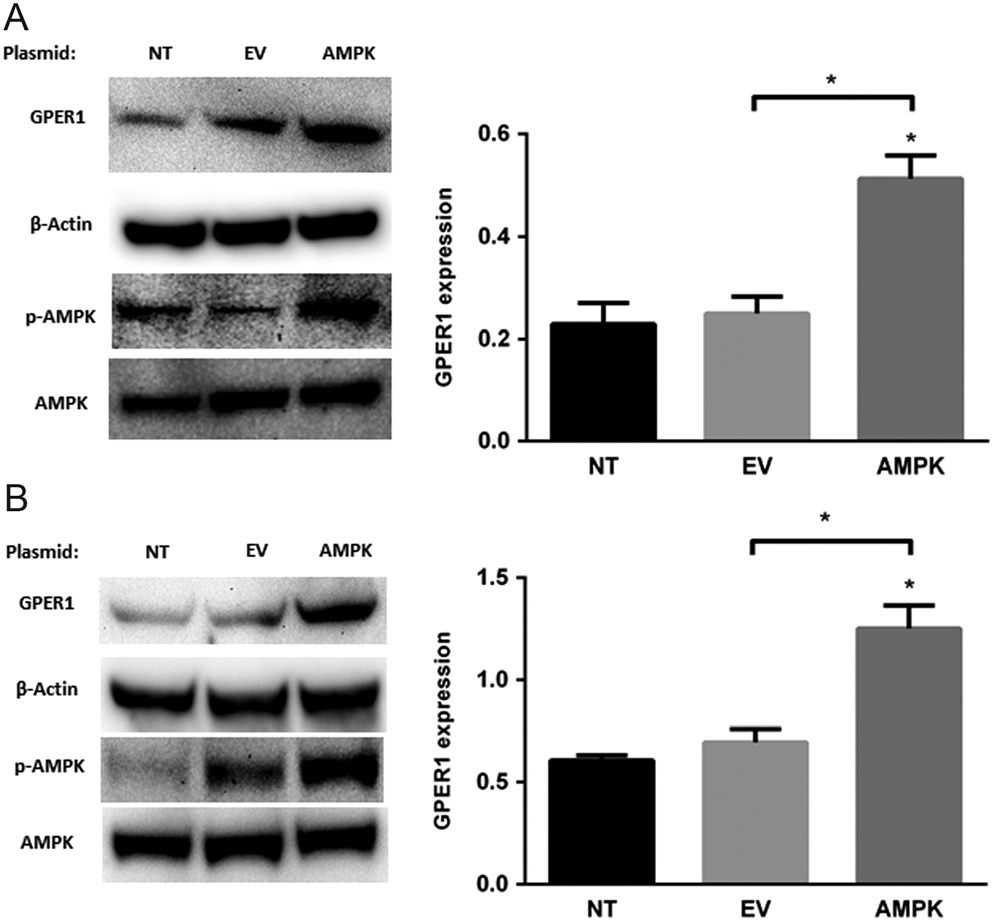
Expression of constitutively active AMPKα induced GPER1 expression in breast cancer cells. Immunoblot analysis of GPER1 expression 24 h post transfection with a plasmid expressing constitutively active AMPKα (aa1-312) in (A) MCF-7 cells and (B) T-47D cells. Results are the average of three independent experiments and GPER1 expression was normalized to β-actin. Error bars are the standard error of the mean and statistical significance (*P* < 0.05) is noted using *.

To provide evidence that the observed [D-glucose]-sensitive modulation of GPER1 expression is dependent on AMPK activation, ELT-3 and ELT-6 cells were treated with the selective AMPK inhibitor dorsomorphin (Compound C) that has previously been shown to inhibit the AMPK in this cell type (Short *et al.* 2008). ELT-3 and ELT-6 cells were treated with vehicle, 1, 2.5 or 5 μM dorsomorphin for 24 h followed by immunoblot analysis of GPER1, pAMPK (Thr 172) and AMPK protein levels. Results from these experiments indicated that all tested doses of dorsomorphin decreased the accumulation of pAMPK (Thr 172) and GPER1 protein levels in both ELT-3 and ELT-6 cells (Fig. 4A and B), while 5 μM dorsomorphin did not alter the GPER expression significantly in ELT-3 cells. To provide additional evidence that the observed increase in GPER1 expression resulting from D-glucose deprivation was AMPK-dependent, MCF-7 and T-47D cells were serum and D-glucose-deprived for 24 h followed by 24 h of treatment with vehicle, 1, 2.5 or 5 μM dorsomorphin. After 24 h of treatment, total RNA and protein was isolated for real-time PCR and immunoblot analysis, respectively. Results show that dorsomorphin treatment reduced the accumulation of pAMPK (Thr 172) and GPER1 protein levels in MCF-7 (Fig. 5A and B) and in T-47D (Supplementary Fig. 4). Additionally, GPER1 transcript levels were significantly reduced upon dorsomorphin treatment in MCF-7 cells (Fig. 5C). Next, breast cancer cells were treated with vehicle, 50, 100 or 200 μM of the specific AMPK activator 5-Aminoimidazole-4-carboxamide ribonucleotide (AICAR) for 24 h in 25 mM D-glucose followed by isolation of total RNA and protein for real-time PCR and immunoblot analysis, respectively. As previously observed, the accumulation of pAMPK (Thr 172) and GPER1 protein levels were relatively low in cells grown in the presence of high [D-glucose]. However, when treated with AICAR, a dose-dependent increase in the accumulation of pAMPK (Thr172) and GPER1 protein levels was observed in MCF-7 and T-47D cells (Fig. 6A and B). GPER1 transcript levels were also increased by AICAR treatment in a dose-dependent fashion in both cell lines (Fig. 6C). Finally, constitutively active AMPK was expressed in MCF-7 and T-47D cells using the mammalian expression vector pEBG‐AMPK α1(1‐312) (Crute *et al.* 1998) followed by immunoblot analysis of GPER1 and pAMPK levels 24 h post transfection. The levels of GPER1 and pAMPK were significantly increased in both MCF-7 and T-47D cells compared to each non-transfected (NT) parental cells and empty vector (EV) transfected cells (Fig. 7). Taken together, these data indicate that the observed increase in GPER1 gene expression and protein levels in D-glucose-deprived cells was AMPK-dependent.

### D-glucose deprivation enhances tamoxifen-induced IGFBP-1 transcription in breast cancer cells

**Figure 8:**
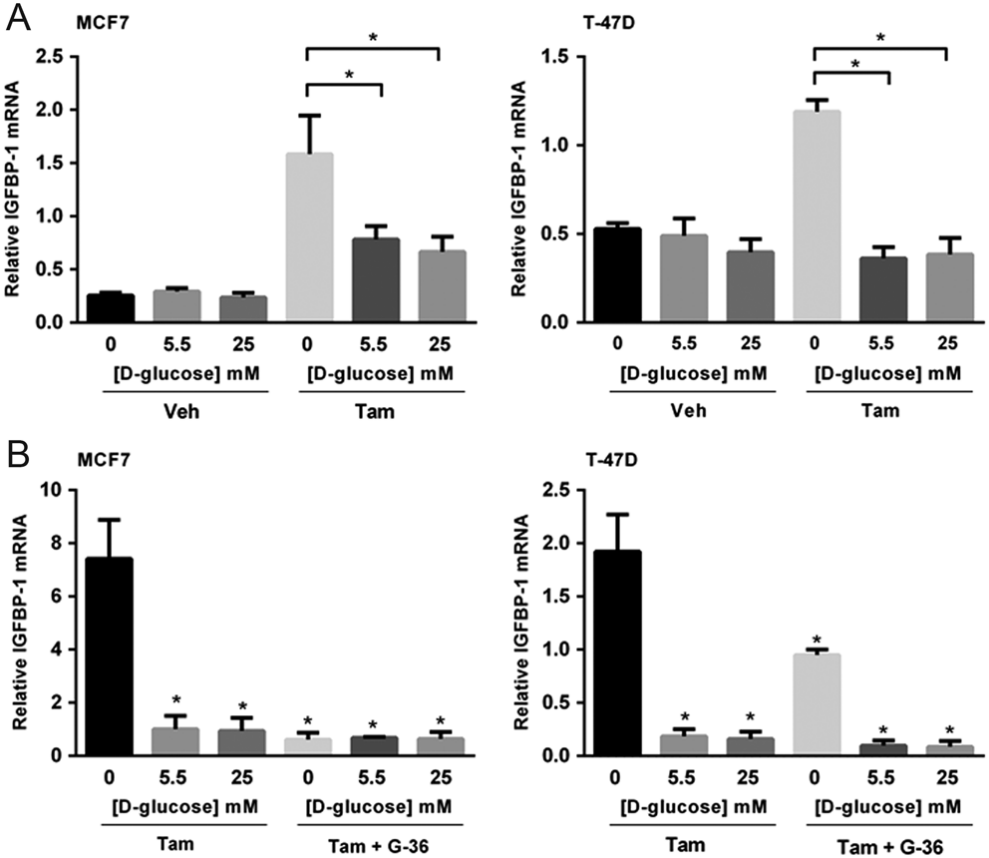
Low D-glucose concentration potentiates IGFBP-1 transcription in Tam-treated breast cancer cells. Quantitative real-time PCR analysis of IGFBP-1 transcript level in breast cancer cells cultured in serum-free DMEM with indicated [D-glucose] after 24-h treatment with (A) 1 µM Tam or (B) 1 µM Tam + 1 µM G-36. Results are the average of three independent experiments and IGFBP-1 transcript levels were normalized using RPL30. Error bars are the standard error of the mean and statistical significance (*P* < 0.05) is noted using *.

We previously reported that 4-hydroxytamoxifen (Tam), the active metabolite of tamoxifen, induces the expression of IGFBP-1 in a GPER1-dependent manner in MCF-7 breast cancer cells (Vaziri-Gohar & Houston 2016). It was reasoned that if GPER1 mediates tamoxifen-induced IGFBP-1 expression and D-glucose deprivation increases GPER1 protein levels, then D-glucose deprivation will enhance IGFBP-1 transcription in Tam-treated cells. To determine if exposure to relatively low [D-glucose] resulted in a significant increase in IGFBP-1 transcription in breast cancer cells, MCF-7 and T-47D cells were treated with 1 μM Tam for 24 h in media containing 0, 5.5 or 25 mM D-glucose and total RNA was isolated for quantitative real-time PCR analysis. While the IGFBP-1 transcript level was not significantly changed by [D-glucose], the transcript levels for IGFBP-1 after Tam treatment were significantly increased in MCF-7 and T-47D cells when cultured in 0 mM D-glucose compared to 5.5 mM and 25 mM D-glucose (Fig. 8A). These data suggest that the [D-glucose] concentration modulates breast cancer cell response to tamoxifen treatment. In order to demonstrate the GPER1 dependence of the observed IGFBP-1 induction, Tam-treated MCF-7 and T-47D cells were co-treated with the GPER1 inhibitor G-36 following a previously described protocol (Vaziri-Gohar & Houston 2016). The addition of G-36 significantly reduced the Tam-stimulated increase in IGFBP-1 transcript level in breast cancer cells cultured in 0 mM D-glucose (Fig. 8B). Furthermore, we show that the level of extracellular IGFBP-1 after Tam treatment in both MCF-7 and T-47D cells is attenuated upon co-treatment with G-36 (Supplementary Fig. 5). These data indicate that the observed enhancement of Tam-induced IGFBP-1 in breast cancer cells cultured in 0 mM D-glucose is dependent on GPER1 and suggest that breast cancer cells exposed to high levels of D-glucose may be less sensitive to some mechanisms of tamoxifen action due to decreased GPER1 expression.

## Discussion

Numerous reports have demonstrated the molecular mechanisms of GPER1-mediated cell signaling in breast cancer cells (Lappano *et al.* 2014) and GPER1 activation by the selective ER modulator tamoxifen has been demonstrated (Vivacqua *et al.* 2012, Li *et al.* 2013, Vaziri-Gohar & Houston 2016, Rouhimoghadam *et al.* 2018), the mechanisms that regulate GPER1 expression are not well understood. Due to the important role that GPER1 has in breast cancer cell biology and treatment outcomes, it is important to understand the molecular determinants of GPER1 expression in this cell type. Recent work reported the loss of glucose homeostasis in GPER1 knockout (GPER1 KO) mice (Martensson *et al.* 2009, Sharma *et al.* 2013) suggesting that GPER1 expression may be sensitive to D-glucose concentrations. Results reported herein reveal that GPER1 expression in breast cancer cells is sensitive to [D-glucose] and requires AMPK-dependent signaling. Furthermore, these data indicated the response of breast cancer cells to tamoxifen treatment varied depending on the [D-glucose] to which the cells were exposed. Taken together, this study revealed a previously unknown mechanism that regulates GPER1 expression in ERα positive breast cancer cells and alters sensitivity to tamoxifen treatment.

D-glucose induces and represses a broad spectrum of genes in many cell lines (Foufelle *et al.* 1998). In addition to the discovery of glucose response elements within the promoter region in glucose-induced genes, high [D-glucose] has been shown to repress gene expression (Foufelle *et al.* 1998, Canto & Auwerx 2010). In the D-glucose-deprived state, AMPK is activated resulting in downstream induction of genes by activation of transcription factors such as FOXO3a (Greer *et al.* 2007), CREB (Thomson *et al.* 2008), p73 (Adamovich *et al.* 2014) and p53 (Jones *et al.* 2005). Interestingly, initial analysis of a 5 kb putative promoter sequence for human GPER revealed potential response elements for FOXO transcription factors (data not shown). Future work aimed at identifying the transcription factors required for the observed increase in GPER1 transcription and expression will provide a more complete understanding of D-glucose sensitive GPER1 regulation (Greer *et al.* 2009, Zhao *et al.* 2011, Sengupta *et al.* 2013) and may lead to the identification of additional markers for sensitivity of breast cancer cells to tamoxifen treatment.

Previously, we showed that 4-hydroxytamoxifen (Tam) induces the expression of insulin-like growth factor-binding protein 1 (IGFBP1) via a GPER1-dependent mechanism in MCF-7 and SkBr-3 breast cancer cells (Vaziri-Gohar & Houston 2016). Based on these data, we hypothesized that [D-glucose]-dependent changes in GPER1 expression alter the IGFBP-1-dependent tamoxifen response of breast cancer cells. While IGFBP-1 expression was not changed when [D-glucose] was varied in MCF-7 breast cancer cells, Tam-dependent IGFBP-1 induction was significantly increased in breast cancer cells when treated while D-glucose-deprived. While [D-glucose] altered the tamoxifen response in both MCF-7 and T-47D breast cancer cells, the IGFBP-1 induction was not exactly the same in both cell lines tested. In T-47D cells, Tam treatment induced IGFBP-1 transcription only in the D-glucose-deprived condition. This difference may provide insight regarding IGFBP-1 expression in Tam-treated cells since IGFBP-1 expression has been shown to be p53-dependent (Leu & George 2007, Chua *et al.* 2015).

Type 2 diabetes is a well-known risk factor for breast cancer (Liao *et al.* 2011, Wang *et al.* 2013, Tabassum *et al.* 2016) and is associated with high blood glucose levels. In the context of the data presented in this contribution, high blood glucose levels may suppress GPER1 expression thus minimizing the regulation of IGF-1-dependent signaling in breast cancer cells by disrupting the relative levels of expression for the multiple ERs compared to the relative levels observed when circulating glucose levels are normal. Furthermore, the role that GPER1 has during tamoxifen treatment could be minimized in patients with high blood glucose levels that are receiving tamoxifen treatment. Interestingly, the type 2 diabetic treatment Metformin synergizes with tamoxifen to inhibit ERα positive breast cancer cell proliferation (Ma *et al.* 2014). This suggests that the blood glucose levels of patients receiving tamoxifen treatment may decrease efficacy. There is much to be understood with regard to the role that [D-glucose] has during the treatment of breast cancer with tamoxifen. Additional studies will provide necessary information to reliably predict which complicating health conditions may impact the efficacy of tamoxifen for breast cancer treatment and if co-treatment with other currently used pharmaceuticals such as metformin are warranted.

## Supplementary Material

Supplementary figure 1. Relative GPER1 expression in MCF-7 and T-47D cells cultured in DMEM containing 25mM D-Glucose and 10% FBS.

Supplementary figure 2. Immunoblot analysis of GPER1 expression in A, MCF-7 and B, T-47D cells when cultured in media containing 10% FBS with the indicated concentration of D-glucose.

Supplementary figure 3-1. Cq values of RPL30 in the indicated qRT-PCR reactions. Results are the average of 3 independent experiments. Error bars are the standard error of the mean and statistical significance (p < 0.05) is noted using *.Supplementary figure 3-2. Cq values of RPL30 in the indicated qRT-PCR reactions. Results are the average of 3 independent experiments. Error bars are the standard error of the mean and statistical significance (p < 0.05) is noted using *.

Supplementary Figure 4. AMPK inhibition decreased GPER1 expression in T-47D cells cultured in low [D-glucose]. A, Immunoblot analysis GPER1 expression and B, Quantitative real-time PCR analysis of GPER1 transcript level in T-47D cells cultured in low [D-glucose] after 24 hour treatment with the indicated dose of dorsomorphin. Quantitative results are the average of 3 independent experiments. GPER1 transcript levels were normalized using RPL30 and Error bars are the standard error of the mean and statistical significance (p < 0.05) is noted using *.

Supplementary figure 5. Accumulation of extracellular IGFBP-1 in breast cancer cell cultures after Tam treatment. Immunoblot analysis of extracellular IGFBP-1 from A, MCF-7 and B, T-47D conditioned media after 24-hour treatment with 1µM Tam or 1µM Tam + 1µM G-36. Coomassie staining was used to assure consistent loading between samples. Results are representative of 2 independent experiments.

## Declaration of interest

The authors declare that there is no conflict of interest that could be perceived as prejudicing the impartiality of the research reported.

## Funding

This work was supported by an Institutional Development Award (IDeA) P20GM103451 and 1SC1GM127175 from the National Institute of General Medical Sciences of the National Institutes of Health.

## Author contribution statement

Designed the experiments: K D H and Y Z. Performed the experiments: Y Z. Analyzed data: K D H and Y Z. Wrote the manuscript: Y Z. Edited the manuscript: K D H and Y Z. K D H and Y Z read and approved the final manuscript.
