# Supplementary figures and images for: Glucose-dependent GPER1 expression modulates tamoxifen-induced IGFBP-1 accumulation
Source: J Mol Endocrinol. 2019 May 29;63(2):103–12. doi: 10.1530/JME-18-0253 (PMC6598863; doi:10.1530/JME-18-0253)

Suppl Figure 1

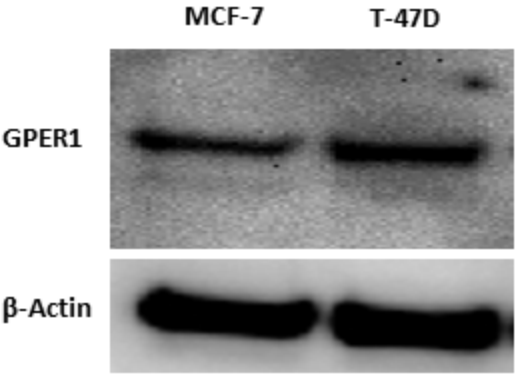

Supplement: Supplementary figure 1. Relative GPER1 expression in MCF-7 and T-47D cells cultured in DMEM containing 25mM D-Glucose and 10% FBS. [file supplementary_figure_1.pdf]

Suppl Figure 2

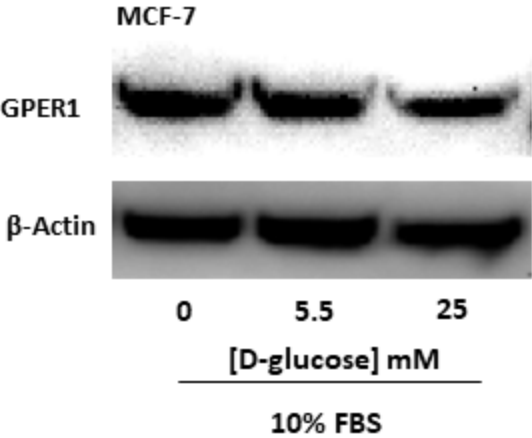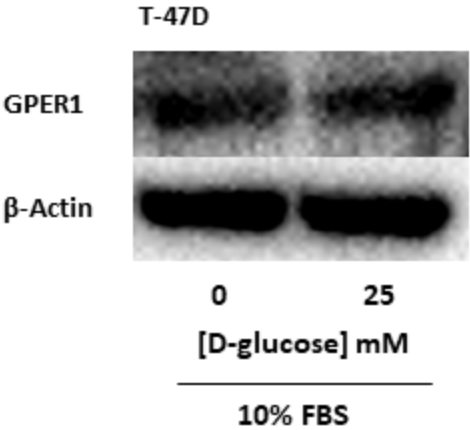

Supplement: Supplementary figure 2. Immunoblot analysis of GPER1 expression in A, MCF-7 and B, T-47D cells when cultured in media containing 10% FBS with the indicated concentration of D-glucose. [file supplementary_figure_2.pdf]

**A**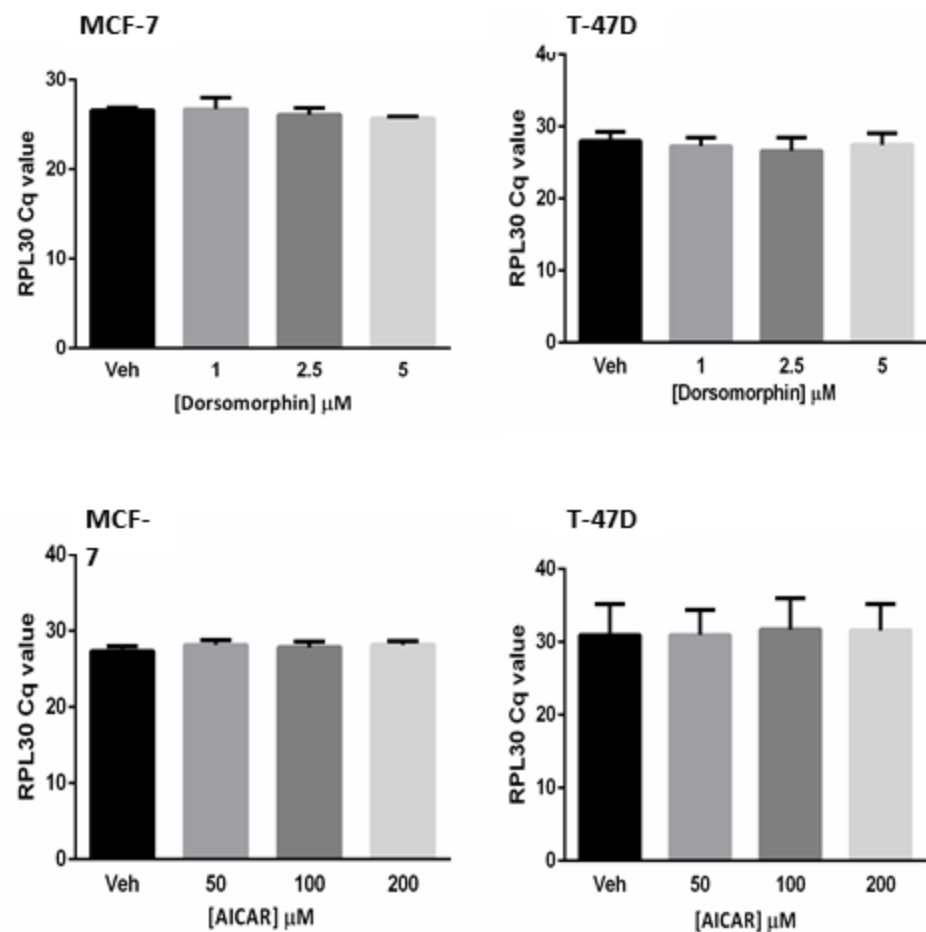**B**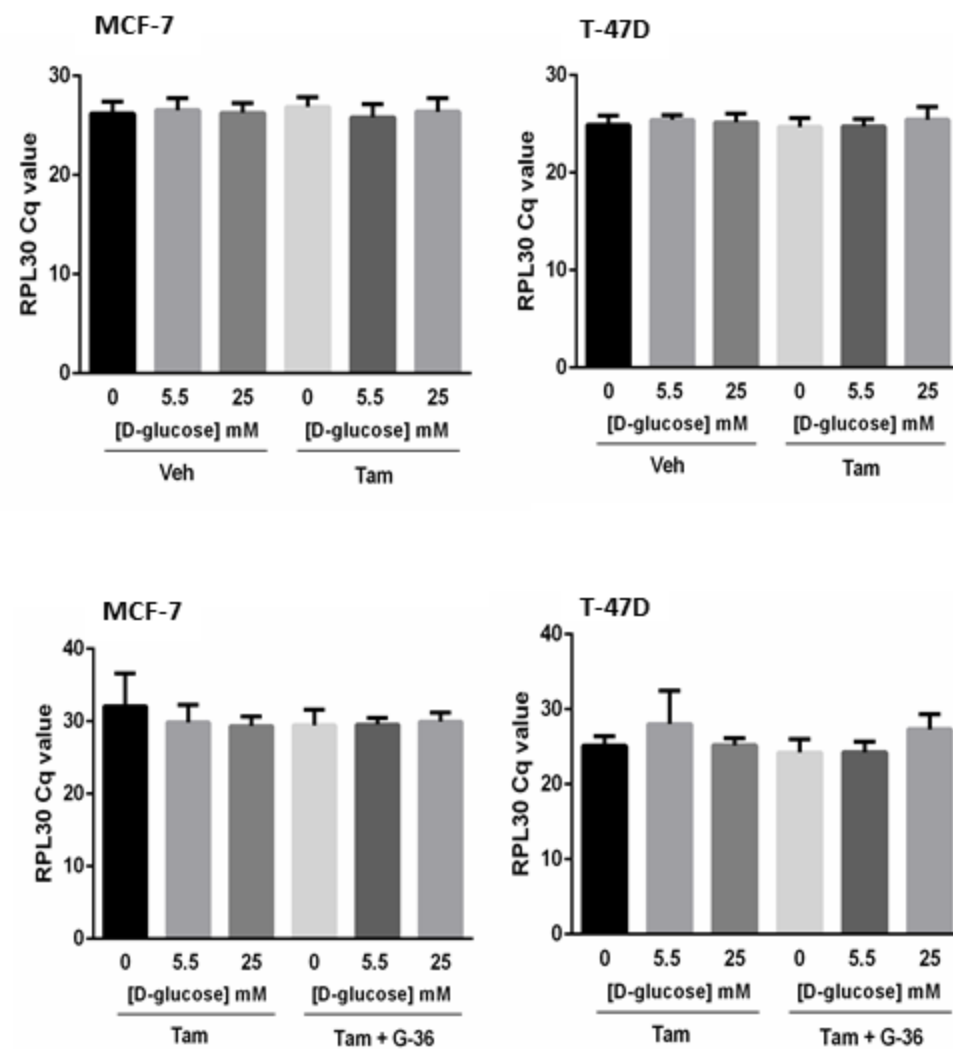

Supplement: Supplementary figure 3-1. Cq values of RPL30 in the indicated qRT-PCR reactions. Results are the average of 3 independent experiments. Error bars are the standard error of the mean and statistical significance (p < 0.05) is noted using *.Supplementary figure 3-2. Cq values of RPL30 in the indicated  [file supplementary_figure_3.pdf]

Suppl Figure4

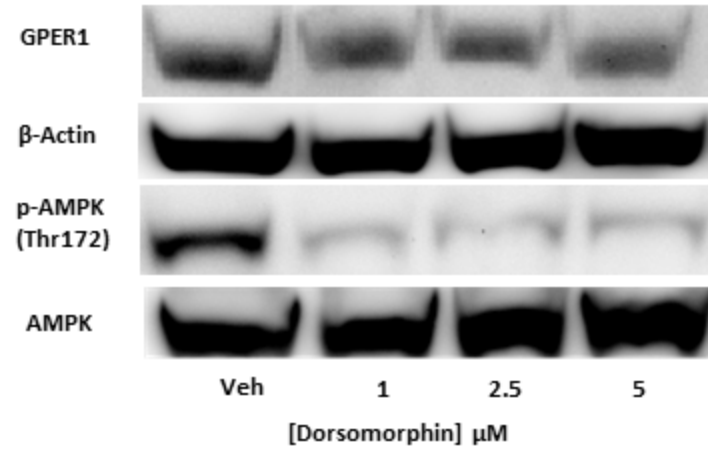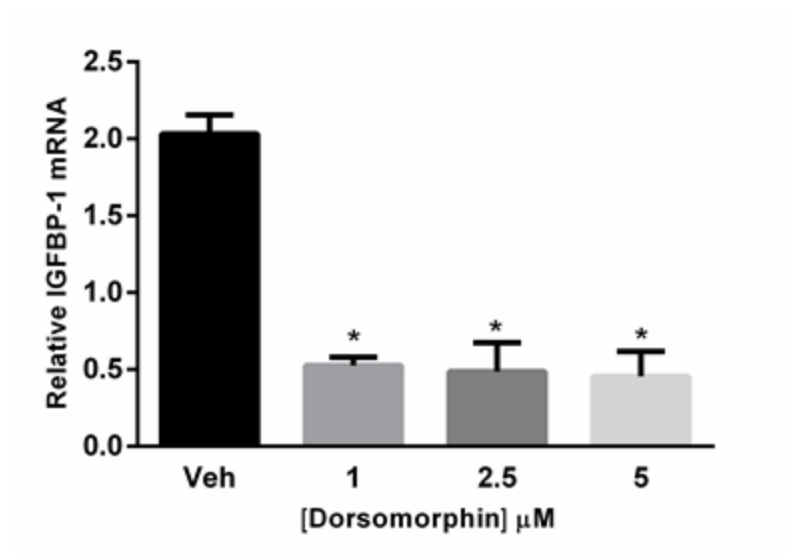

Supplement: Supplementary Figure 4. AMPK inhibition decreased GPER1 expression in T-47D cells cultured in low [D-glucose]. A, Immunoblot analysis GPER1 expression and B, Quantitative real-time PCR analysis of GPER1 transcript level in T-47D cells cultured in low [D-glucose] after 24 hour treatment with the indi [file supplementary_figure_4.pdf]

Suppl Figure5

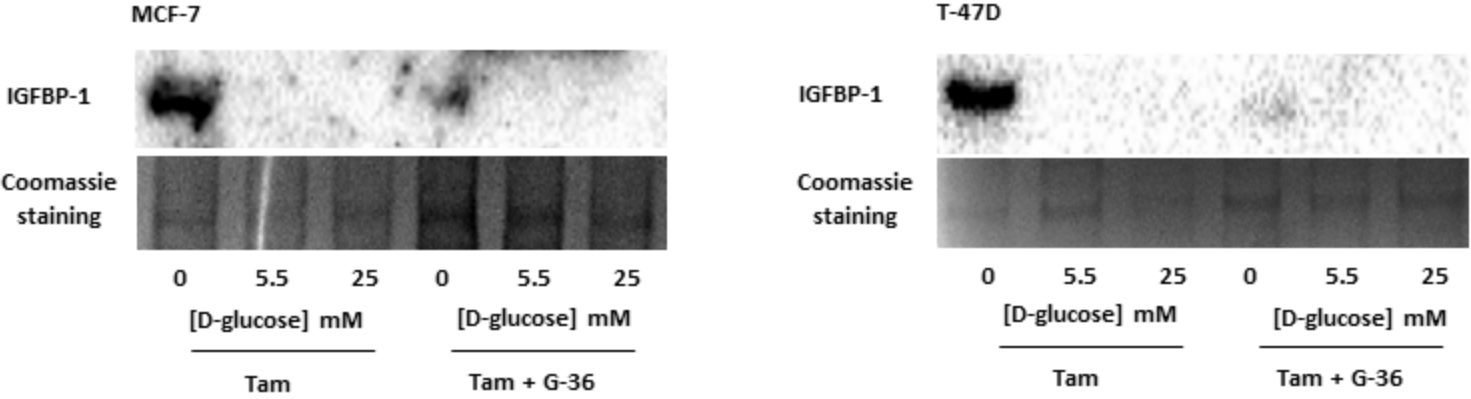

Supplement: Supplementary figure 5. Accumulation of extracellular IGFBP-1 in breast cancer cell cultures after Tam treatment. Immunoblot analysis of extracellular IGFBP-1 from A, MCF-7 and B, T-47D conditioned media after 24-hour treatment with 1µM Tam or 1µM Tam + 1µM G-36. Coomassie staining was used to assur [file supplementary_figure_5.pdf]
